# Résection endoscopique des polypes colorectaux pédiculés en utilisant un lasso largable au fil catgut chromé: une alternative a la polypectomie conventionnelle? A propos d'une série de cas

**DOI:** 10.11604/pamj.2014.18.14.3899

**Published:** 2014-05-03

**Authors:** Firmin Ankouane, Dominique Noah Noah, Bernadette Ngo Nonga, Michèle Tagni-Sartre, Gabriel Modjo, Elie Claude Ndjitoyap Ndam

**Affiliations:** 1 Département de Médecine Interne et Spécialités, Faculté de Médecine et des Sciences Biomédicales, Centre Hospitalier et Universitaire de Yaoundé, Université de Yaoundé I, Cameroun; 2 Faculté de Médecine et des Sciences Pharmaceutiques, Université de Douala, Hôpital Central de Yaoundé, Cameroun; 3 Département de Chirurgie et Spécialités, Centre Hospitalier et Universitaire de Yaoundé, Université de Yaoundé I, Cameroun; 4 Service d'Hépato-Gastroentérologie, Centre Médical la Cathédrale, Yaoundé, Cameroun; 5 Département de Médecine Interne et Spécialités, Hôpital Général de Yaoundé, Université de Yaoundé I, Cameroun

**Keywords:** Polypes coliques, catgut, polypectomie endoscopique, coloscopie, endoloop, Cameroun, Afrique subsaharienne

## Abstract

L'intérêt de l'endoscopie dans la résection des polypes colorectaux a été rapporté dans plusieurs études. Les techniques de résection endoscopique sont multiples et maîtrisées dans les pays occidentaux. La technique de mucosectomie endoscopique et celle de la pose d'une anse largable en nylon (endoloop) ont élargi le champ des lésions résécables par endoscopie. Toutefois, malgré cette évolution, la vulgarisation de la polypectomie n'est pas effective. En Afrique subsaharienne, la prise en charge de ces polypes de grande taille nécessite souvent une intervention chirurgicale à ciel ouvert ou une évacuation sanitaire onéreuse dans un pays en Occident. Nous rapportons une nouvelle approche de polypectomie endoscopique des polypes pédiculés colorectaux, en utilisant un lasso largable au fil catgut chromé 2/0. Les polypes pédiculés étaient situés soit au niveau du sigmoïde soit au rectum. Après avoir passé le lasso autour du pédicule, le n'ud du lasso est serré autour de celui-ci pour strangulation. En moyenne 6 jours après la procédure, le polype est récupéré dans les selles. Une colonoscopie de contrôle est nécessaire pour confirmer la résection du polype. Cette technique peu coûteuse et accessible, devrait être vulgarisée dans les pays en voie de développement avec des plateaux techniques pauvres. Elle a ses limites et ses inconvénients qui doivent être connus de l'opérateur.

## Introduction

L'endoscopie moderne permet le diagnostic et l'exérèse complète de nombreux polypes colorectaux. L'exérèse endoscopique est souvent suffisante pour les polypes invasifs s'ils sont pédiculés. Les techniques de résection endoscopique sont multiples et maîtrisées dans les pays occidentaux [1–3]. La technique de mucosectomie endoscopique et celle de la pose d'une anse largable en nylon (endoloop) ont élargi le champ des lésions résécables par endoscopie [4, 5]. Toutefois, malgré cette évolution de la polypectomie endoscopique, sa vulgarisation n'est pas effective [6, 7]. En Afrique subsaharienne la prise en charge thérapeutique de ces polypes de grande taille nécessite souvent une intervention chirurgicale à ciel ouvert ou une évacuation sanitaire onéreuse dans un pays en Occident. Cette évacuation est la conséquence d'un manque de matériel adéquat [6].

Nous rapportons un cas de résection de polype pédiculé colorectal, réalisée au Centre Médical la Cathédrale à Yaoundé en utilisant un lasso largable au fil catgut chromé 2/0, nous expliquons la procédure de pose et présentons un les résultats des autres cas pris en charge de la même façon.

## Méthodes

**Figure 1:**
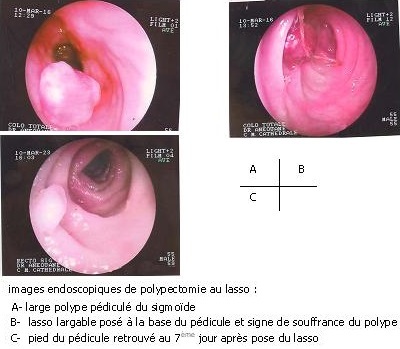
Images endoscopiques de polypectomie au lasso largable

Un homme âgé de 55 ans, hospitalisé pour des rectorragies s’était présenté dans notre service pour une coloscopie. Son histoire remontait à deux ans, date à laquelle il avait remarqué du sang rouge dans les selles sans trouble du transit ni douleur abdominale. Six mois plus tôt il aurait présenté une rectorragie abondante qui avait nécessité une transfusion sanguine de 2 poches de sang complet. Ses antécédents personnels et familiaux étaient sans particularité. Son examen clinique y compris le toucher rectal était normal. Son bilan biologique révélait uniquement une anémie sévère à 6 g% d′hémoglobine nécessitant une nouvelle transfusion sanguine. Une première coloscopie avait mis en évidence un polype pédiculé du sigmoïde de 2 cm environ avec un pédicule long et large (Figure 1 - A). Des biopsies de la tête du polype n'ont pas été effectuées. Une deuxième coloscopie a permis la pose d'un lasso largable au fil catgut chromé 2/0 autour du pédicule (Figure 1 - B). Les suites immédiates ont été simples. Le patient a récupéré le polype dans les selles au 5ème jour post endoscopie, ce qui nous a permis de réaliser une coloscopie de contrôle au 7^ème^ jour confirmant la résection du polype (Figure 1 - C). Avec un recul de 10 mois, le malade n′a pas présenté de récidive hémorragique.

### Technique de pose du lasso largable par endoscopie

**Figure 2:**
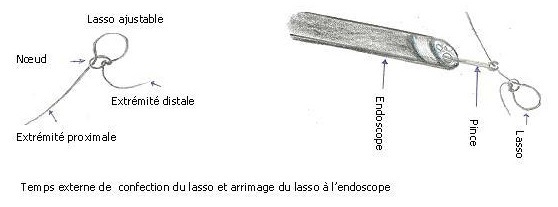
Temps externe de confection du lasso et arrimage du lasso à l'endoscope

1^er^ temps (externe): Confection du lasso au fil catgut chromé 2/0 (Figure 2)

Le lasso, d'un diamètre ajustable à adapter au polype, est réalisé avec un n'ud chirurgical dit simple à un tour. Il est laissé aux extrémités du lasso assez de fil pour une longueur suffisante permettant les manipulations au moment de la pose. Par le fibroscope, une pince classique à biopsie est passée par le canal d'aspiration jusqu’à l'extrémité du fibroscope. L'extrémité proximale du lasso y est fixée fermement par l'intermédiaire de la fermeture de la pince. Le lasso est ajusté et gardé bien ouvert. Afin d’éviter les traumatismes, la pince est légèrement retirée à l'intérieur du canal au moment de l'introduction du fibroscope.

**Figure 3:**
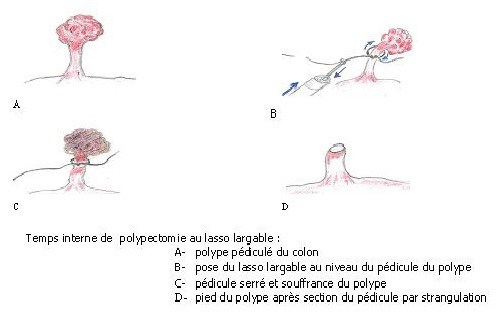
Temps interne de polypectomie au lasso largable

2^ème^ temps (interne): Pose du lasso largable au pédicule du polype pour étranglement (Figure 3)

Une fois introduit à vue dans la lumière colique, le fibroscope est dirigé jusqu'au polype. La pince est maintenue fermée par l'aide opérateur durant la man'uvre. La pince est ressortie au niveau du site du polype sans l'ouvrir. Le lasso est passé autour de la tête du polype jusqu’à un niveau suffisant et visible du pédicule. Par deux mouvements en sens inverse l'un de l'autre, le fibroscope avançant pendant que la pince recule dans le canal, le n'ud du lasso est régulièrement serré autour du pédicule. En fin de procédure, le lasso est largué par l'aide opérateur.

## Résultats

Durant la période de 6 mois allant de janvier à juin 2010, cinq patients porteurs de polypes pédiculés du colon ou du rectum ont bénéficié d'une polypectomie endoscopique par lasso largable au catgut chromé 2/0. Il s'agissait de 3(60%) hommes et de 2(40%) femmes, d’âge moyen de 42,5ans (extrêmes 23 et 55ans). Les symptômes principaux ayant conduit au diagnostic de polype étaient les rectorragies et l'anémie ferriprive chez tous les patients, dont un transfusé. Selon la localisation des polypes, 60% (3/5) étaient situés dans le sigmoïde et 40% (2/5) dans le rectum. La taille moyenne des polypes était de 2,1cm (1-3cm). Des polypes récupérés dans les selles, 03(60%) polypes ont été analysés à l'histologie, dont 02 adénomes tubuleux et 01 polype hyperplasique. Les autres polypes (02) étaient non analysables à l'histologie de part leur état. Le délai moyen de chute du polype étranglé était de 6 jours (4-10jours) et la durée moyenne de suivi des patients de 4,1 mois (extrêmes 1 et 10 mois) Tableau 1.

**Tableau 1:** Profil des patients, caractéristiques des polypes, localisation et délai de chute du polype

N°	Age (ans)/genre	Type de polype	Taille du polype (cm)	Localisation du polype	Histologie du polype	Délai de chute en (j)	Durée du suivi en mois*
1	55/M	Pédiculé	2	Sigmoïde	non	5 ^a^	10
2	23/M	Pédiculé	3	Rectum	Adénome tubuleux	8 ^b^	2
3	38/F	Pédiculé	2,5	Sigmoïde	Adénome tubuleux	10^a^	1
4	45/M	Pédiculé	1	Rectum	non	4^b^	4
5	52/F	Pédiculé	2	sigmoïde	Polype hyperplasique	7^a^	3,5

cm : centimètres, j : jours.

a Contrôle par rectoscopie rigide

b Contrôle par sigmoïdoscopie flexible.

* Suivi des symptômes ayant suscités la consultation, pour les cinq patients il s'agissait des rectorragies et d'anémie ferriprive.

## Discussion

Plusieurs techniques de résection endoscopique de polypes colorectaux ont été décrites dans la littérature [2]. Nos observations illustrent bien que la résection de polypes pédiculés en utilisant le fil catgut chromé 2/0 est une alternative peu coûteuse et efficace quand les techniques conventionnelles ne sont pas accessibles comme dans certains pays en Afrique Subsaharienne [6]. A notre connaissance, hormis la technique d'hémostase endoscopique, avant polypectomie, à l'aide d'une anse largable en nylon (endoloop) décrite pour la première fois en 1989 par Hachisu [8, 9], aucune autre technique similaire n'a été rapportée dans la littérature.

La décision de résection endoscopique du polype en utilisant le lasso largable au fil catgut doit tenir compte des nécessités matérielles et techniques, de l'habileté de l'opérateur, de la taille et de la localisation du polype. Mais aussi, de l’état général et des tares du patient. Nous avons effectué la polypectomie sur des polypes recto-sigmoïdiens pédiculés, lésions fréquentes dans notre pratique hospitalière [7], chez des patients en bon état général. Tous les patients n'avaient aucune contre indication à la polypectomie conventionnelle.

Une bonne connaissance de la classification endoscopique de Paris des polypes [10–13] est nécessaire pour déterminer les polypes à réséquer par lasso largable au catgut chromé. Le fil catgut chromé 2/0 présente des particularités adaptées au milieu colique par sa résistance, sa maniabilité et le fait de garder un lasso maniable pendant la procédure. L'utilisation d'un autre fil, moins déformable est une alternative.

L'hémorragie est une complication redoutée pendant la procédure, par suite d'une section accidentelle du pédicule ou lors de la chute d'escarre, comme dans la technique conventionnelle [14]. Toutefois, plusieurs études ont montré que l'anse largable en nylon (endoloop) du fait de l’étranglement du pédicule et l'injection d'adrénaline sont supérieures à la polypectomie conventionnelle dans la prévention du risque hémorragique [15]. Autre inconvénient et non le moindre l'absence parfois, de l'examen histopathologique du polype réséqué pour déterminer le caractère complet ou non de la résection endoscopique, élément nécessaire pour décider entre une surveillance endoscopique et une chirurgie complémentaire [16, 17]. Enfin, la multiplication des examens endoscopiques de contrôle a un coût, limitant la technique. Néanmoins, ce coût reste raisonnable par rapport à une évacuation sanitaire.

## Conclusion

Plusieurs techniques validées sont disponibles pour la résection endoscopique des polypes colorectaux. Cependant, leur coût financier et leur maîtrise les rendent inaccessibles pour bon nombre de malades dans le monde, particulièrement dans les pays en voie de développement. La technique de résection endoscopique par lasso largable au catgut chromé mérite une attention particulière. Cette attention doit être dans le sens d'apports scientifiques complémentaires pour son amélioration et sa vulgarisation.
